# The Development and Prediction of Athletic Performance in Freestyle Swimming

**DOI:** 10.2478/v10078-012-0027-3

**Published:** 2012-05-30

**Authors:** Arkadiusz Stanula, Adam Maszczyk, Robert Roczniok, Przemysław Pietraszewski, Andrzej Ostrowski, Adam Zając, Marek Strzała

**Affiliations:** 1 The Jerzy Kukuczka Academy of Pchysical Education, Katowice, Poland.; 2 University School of Physical Education, Cracow, Poland.

**Keywords:** swimming, world records, rate of improvement, performance

## Abstract

This paper analyses the dynamics of changes between the performances of elite freestyle swimmers recorded at particular Olympic Games. It also uses a set of chronologically ordered results to predict probable times of swimmers at the 2012 Olympic Games in London. The analysis of past performances of freestyle swimmers and their prediction have revealed a number of interesting tendencies within separately examined results of men and women. Women’s results improve more dynamically compared with men’s. Moreover, the difference between women’s and men’s results is smaller, the longer the swimming distance. As both male and female athletes tend to compete more and more vigorously within their groups, the gap between the gold medallist and the last finisher in the final is constantly decreasing, which provides significant evidence that this sport discipline continues to develop.

## Introduction

Competitive swimming performances have demonstrated consistent and significant improvement over the past 5 decades. The reasons for such improvement are many but are due in part to advanced training procedures, sophisticated selection methods, superior stroke mechanics, standardization and changes in swimming regulations (depth of pool, types of lane lines used, height and angle of starting blocks, water temperature), increased access to the sport, or new swimwear technologies (Arellano et al., 1994; Chatterjee and Laudato, 1996; Costa et al., 2010; O’Connor and Vozenilek, 2011). Considering that the summer Olympic Games are the premier event on the international swimming calendar and that swimmers undertake training and competition in 4-year cycles, with the production of their best performance at the Olympics, their Olympic achievements provide a reliable indication of the development of this sport discipline. A long-held view is that leading swimmers make progress in performance to swim the fastest at the Olympics compared with their earlier performances in the competition year (Arellano et al., 1994; Pyne et al., 2004; Trewin et al., 2004; Heazlewood 2006). It has encouraged a number of researchers to attempt to predict future performances by deriving and applying mathematical statistical models based on past performances in athletics (Pendergast, 1990; Heazlewood and Lackey 1996; Edelman-Nusser et al., 2002; Busso and Thomas 2006; Heazlewood, 2006; Silva et al., 2007; Anderson et al., 2008).

While sport performance analysis mainly consists in observing and then trying to explain trends that have been identified in the chosen period of time, a forecasting activity aims to provide coaches and athletes with information on the likely, future performances in the given sport discipline. This information may help coaches define realistic goals and training methods (Pyne et al., 2004), describe and estimate the progression and the variability of performance during and between seasons, find hypothetical chronological points to predict swimmer’s performance throughout his or her career or in a given time frame, and determine the probability that the swimmer will reach finals or win medals in important competitions (Hellard, et al., 2005; Costa et al., 2010).

To find an explanation why as many as 43 world swimming records were broken in 2009, O’Connor and Vozenilek (2011) have analysed athletes’ performances in two different sports: swimming and track running. They assumed that the two sports, dissimilar because of the structure of movements, but mainly due to the training and competition environment, share some key similarities from the perspective of sport performance analysis, such as the duration of the effort and the resource of energy necessary for working muscles. Consequently, the training methods they use are also similar. The researchers have concluded that the most probable reason why swimmers were so successful was modern swimwear completely covering their bodies.

Chatterjee and Laudato (1996) who have analysed athletes’ performances recorded over a long period of time have found very interesting facts about the relations between men’s and women’s results. Their overall conclusions can be stated as follows: women’s rates of improvement, defined technically, are increasing whereas for most men’s events the corresponding rates are slowing down. In explaining this tendency the authors point to greater participation of women in professional sport and to the growing interest of scientists in giving support to coaches and female athletes.

Many researchers are pondering over whether at some point of time women will have the same results in sport as men. Although our present knowledge of human anatomy and physiology questions this scenario, a mathematical standpoint underpinned by calculations based on the long-term performances of both male and female athletes makes this situation probable. Heazlewood and Lackey (1996) have constructed a forecasting model showing that at the 2060 Olympic Games female 100 m runners will improve the ratio between their and men’s times, as the average times of the finalists have been estimated at 9.58s for men and 9.57s for women.

This paper analyses the dynamics of changes between the performances of the world-class freestyle swimmers recorded at particular Olympic Games. It also uses a set of chronologically ordered results to forecast the probable times of swimmers at the 2012 Olympic Games in London.

## Material and Methods

### Events

The results of male and female finalists in each freestyle event (50 m, 100 m, 200 m, 400 m, 800 m, 1500 m) held at the Olympic Games between 1896 and 2008 were compiled from Internet resources (www.wikipedia.org, 2012). An exception is the women’s and men’s 50 m and 200 m events introduced, respectively, in 1988 (Seoul Olympic Games) and 1968 (Mexico), as well as the women’s 800 m, which also premiered in Mexico.

The 50 m freestyle is the newest freestyle event that became part of the Olympic program in Seoul in 1988. The 50 m swimmers compete not only for medals, but also for the title of the fastest swimmer in the Olympic Games. The men’s 100 m freestyle event was inaugurated at the first modern-era Olympic Games in Athens in 1896 and since then has been part of all swimming competitions. Women started to race at that distance sixteen years later, in 1912. The long-distance events have been held at the Olympic Games since 1908. The 1500 m event has always been a men’s event. The 1986 Olympic Games mark the beginning of women’s 800 m races.

### Statistical analysis

In order to analyse the improvement in athletes’ performance in a given freestyle event, each time the constant-base and variable-base indices were calculated for the results of eight Olympic Games finalists, while the trend function was selected afterwards. Because a time-series model is a descriptive dynamic model (i.e. one omitting the causes of the course of the analysed phenomenon), the constructed models were verified by testing the significance of the structural parameters (the t-Student test) and the randomness of residuals, as well as the error term autocorrelation (the Durbin-Watson test). The coefficients of convergence indicating the goodness-of-fit between the constructed models and the empirical data were calculated for all swimming events. Swimmers’ performances at the 2012 Olympic Games in London were predicted using the moving average method and linear and non-linear regressions. The regression models’ goodness-of-fit was estimated with the coefficient of determination.

## Results

### 50 m freestyle

**Graph 1:**
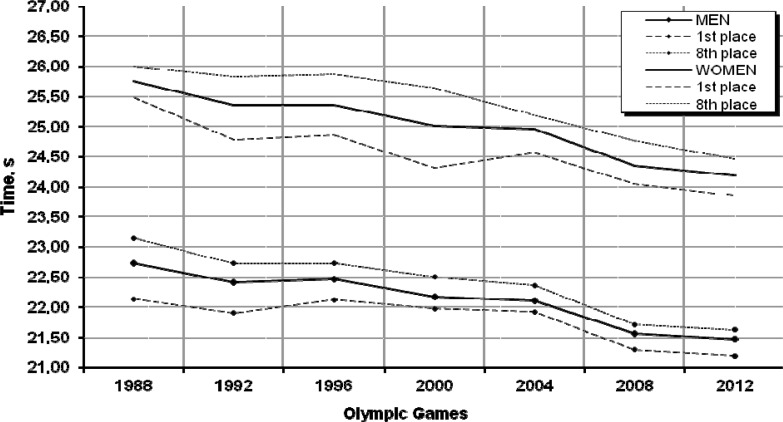
Women’s and men’s performances in the 50 m freestyle during the 6 past Olympic Games and the prediction for London 2012

The data used to produce Graph 1 reveal that the times of both male and female swimmers tend to improve. This tendency is not constant, though, as indicated by their slight regression at the 1996 Olympic Games in Atlanta, amounting to 1.5% for women and 1.6% for men. During the 20 analysed years the eight female and male finalists examined here improved their results, on average, by 1.55 s (6.0%) and 1.28 s (5.5%), respectively. Another interesting finding is that women’s results were systematically moving closer to men’s, as confirmed by the differences between the average women’s and men’s times recorded at the Seoul Olympic Games (3.01 s ) and in Beijing (2.75 s). Moreover, the men’s performance curve plotted in the graph clearly shows that the finalists’ results tend to concentrate, which follows from the rising level of athletic performance, as well as from the growing competition among elite sprint swimmers.

The next step in the analysis was an attempt to predict the results for the upcoming Olympic Games. The trend function was selected based on moving averages and then its goodness-of-fit to the empirical data was determined. The coefficients of convergence *φ*^2^ were 0.11 and 0.06 for women and men, respectively, thus proving that the goodness-of-fit between the linear trend function and the empirical data was very high – the trend function could not explain only 11.0% and 6.0% of variability in athletes’ performance. Therefore, because the trend function was defined well, the probable times of the female and male finalists may be 24.15 s and 21.46 s, respectively. Similar results were obtained from the linear and non-linear regression models. Detailed predictions are shown in table 1.

### 100 m freestyle

**Graph 2:**
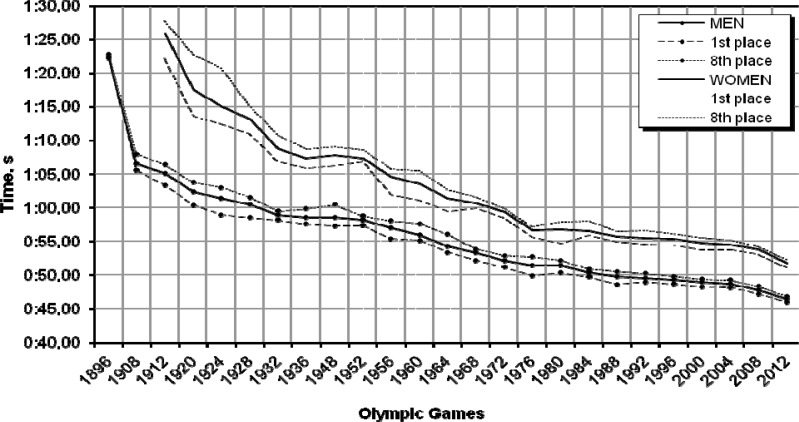
Women’s and men’s performances in the 100 m freestyle during the past 24 Olympic Games and the prediction for London 2012

In Graph 2, which shows a constant improvement of performances of both women and men there are two prominent periods with large increases: one between 1896 and the Berlin Olympic Games (1936), and the other between the Helsinki (1952) and Montreal Olympic Games (1976). Because in the first period swimming events could not be consistently provided with the same technical conditions of competition, our analysis starts in 1952, when the first attempts to standardise the conditions were made and when the Swiss Omega company introduced its quartz time recording system.

The indices calculated for the eight finalists indicate that between 1952 and 2008 the performances of the 100 m freestyle swimmers showed wave-like progression. At the same time, the variable-base indices reveal small irregularities in the dynamics of the process. Compared with their results achieved at the previous Olympics, male swimmers improved their performances the most in 1964 (3.0%), 1972 (2.0%) and at the Beijing Olympic Games in 2008 (2.8%). The constant-base indices show that in the analysed period the total improvement in their results was 10.84 s (18.6%). A similar trend was found among female swimmers. In their case the improvements were 4.1% in 1956, 3.3% in 1964, and as much as 4.6% in 1976, with the total progression in the analysed period amounting to 13.58 s (20.1%).

The diminishing difference between female and male athletes is also confirmed by a greater rate of improvement in female swimmers. While the 1952 difference between male and female gold medallists was 9.40 s, by 2008 it dropped to 5.91 s.

The prediction of results for the 100 m freestyle event at the 2012 London Olympic Games (table 1) based on time-series and the regression models indicate that women will achieve a time of 51.46 s, while men will swim the distance of 100 m in 46.19 s.

These times may be considerably overestimated, because notwithstanding the models’ high goodness-of-fit (the *φ*^2^ coefficients for women and men were 0.17 and 0.06, respectively), the 2011 and 2012 rankings do not promise such high results at this distance.

### 200 m freestyle

**Graph 3:**
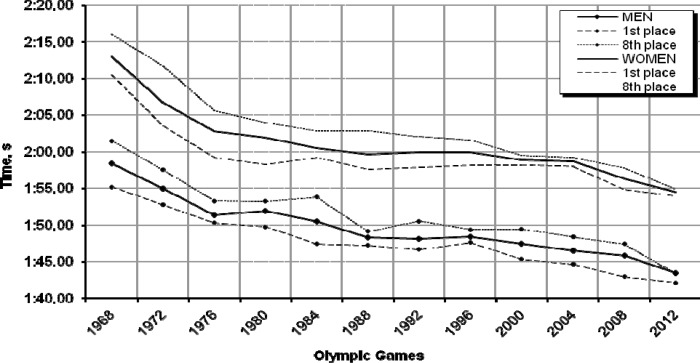
Women’s and men’s performances in the 200 m freestyle during the past 11 Olympic Games and the prediction for London 2012

The analysis of swimmers’ performance in the 200 m freestyle event (Graph 3) shows its wave-like progression among both women and men. Regarding female swimmers, their results increased the most at the Olympic Games in Munich (1972), Montreal (1976) and Beijing (2008) – by 4.7%, 3.1% and 2.6%, respectively. A similar trend was found for the group of male swimmers, where the largest improvements were recorded at the Olympic Games in Munich (1972), Montreal (1976) and Seoul (1988) – by 4.2%, 3.2% and 2.0%, respectively.

The total improvement in performance between the Olympic Games where the event was held for the first time (Mexico 1968) and the most recent Olympic Games in Beijing (2008) was 13.1% for women (17.38 s) and 12.3% for men (14.72 s). When the times of the 200m freestyle swimmers are examined in terms of athletes’ sex, then the differences between them become quite insignificant considering the distance.

For example, the difference between male and female gold medallists at the Mexico Olympic Games (1968) was 15.30 s, which decreased to 11.86 s in Beijing (2008). According to the 2012 prediction for the 200 m event (table 1), in the upcoming Olympic Games women will reach a time of 1:54.25 min and men will swim the distance of 200 m in 1:43.24 min. The likely times of the gold medallists are 1:53.87 min. and 1:42.01 min for women and men, respectively. However, the prediction’ high goodness-of-fit is not high, either for the regression models or the trend based on time-series.

### 400 m freestyle

**Graph 4:**
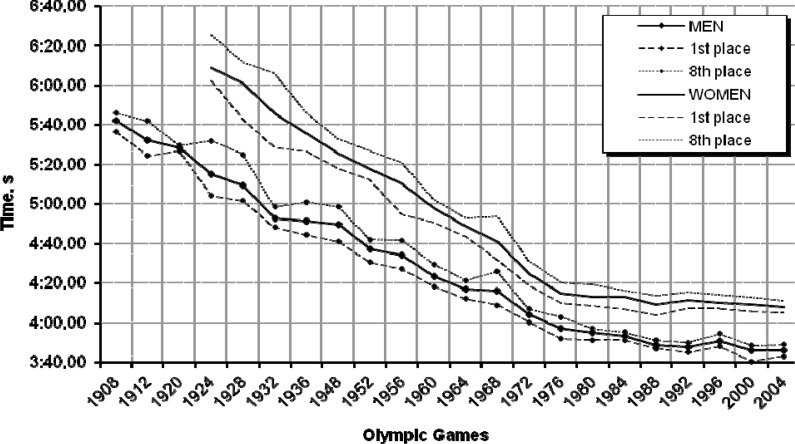
Women’s and men’s performances in the 400 m freestyle during the past 22 Olympic Games and the prediction for London 2012

The performance of the 400 m freestyle swimmers also improved throughout the years (graph 4). The greatest changes were recorded for both women and men at the Olympic Games in Rome (1960), Munich (1972) and Montreal (1976); they amounted, respectively, to 4.0%, 5.9% and 3.6% for women, and 3.9%, 4.5% and 3.0% for men. However, some small symptoms of regression could also be seen in the analysed period: −0.9% for women at the Barcelona Olympic Games (1992) and −1.2% for men at the Atlanta Olympic Games (1996). The curves in graph 4 representing the performances of male and female swimmers additionally show declining differences between the gold medallists and the last finishers in the finals – a proof of growing and balanced competition among the finalists. The trend showing that women’s results are coming closer to men’s is equally distinct. For example, while at the Helsinki Olympic Games (1952) the difference between male and female medallists was 41.40 s, at the Beijing Olympic Games (2008) it decreased by half to 21.36 s.

The 2012 prediction for the 400 m event derived from the results of the Olympic Games finalists recorded since 1952 indicates that the performances of men and women will continue to improve. The female and male finalists’ times may average 3:47.19 min and 3:35.62 min, respectively. That these results are probable is confirmed by the relatively high coefficient of convergence calculated for the regression models and the time series (Table 1).

### 800 m and 1500 m freestyle

**Graph. 5:**
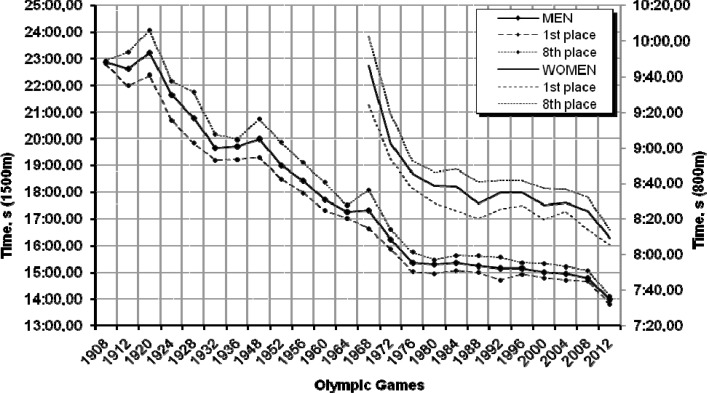
Women’s and men’s performances in the 800 m and 1500 m freestyle during the past 23 Olympic Games and the prediction for London 2012

Analysing the improvement in women’s 800 m results and men’s 1500 m results shown in graph 5 we find a dynamic progression of men’s results until 1976, whereas in female swimmers a similar trend continued to the Moscow Olympics in 1980.

The next Olympic Games where, although the trend was still rising, the performances of both male and female swimmers showed a less dynamic improvement, and where the recorded results were even lower than before, were held in Barcelona (1992) and Athens (2004) (women’s results decreased by −1.2% and − 0.2%, respectively), and in Mexico (1968), Los Angeles (1984) and Atlanta (1996) (men’s results dropped by − 0.2%, − 0.4% and − 0.2%, respectively). In the analysed period women’s performance in the 800 m event improved by 86.01 s in total (14.7%). Men’s results recorded between the Helsinki (1952) and the Beijing Olympic Games (2008) improved by 258.00 *s* (22.6%). The time difference between the gold medallist and the last finisher in the final was decreased in both male and female events. Regarding men, it decreased almost four times, from 83.01 s at the 1952 Olympic Games to 24.28 s in 2008; in the case of women it declined from 38.50 s at the 1968 Olympics to 18.25 s in 2008, thus becoming smaller by more than half.

The 2012 prediction shows that the two events may bring new world records. The estimates are 8:08.10 min for women’s 800 m and 13:59.03 min for men’s 1500 m. Although the coefficient of determination for the regression model constructed to predict the 1500 m time is not low (explaining 86% of the model’s fit), the time seems difficult to achieve.

## Discussion

Because of the multitude of styles and distances, swimming is a sport that ranks second for the greatest number of events held during the Olympic Games. This multitude makes swimmers specialize in particular events and styles which has an impact on their performance. However, the bar is placed so high that few swimmers may hope for winning medals in events involving different swimming styles or distances (Aspenes et al., 2009; Pyne et al., 2004). Another noteworthy fact is that the constant improvement in sport performance has been due to new achievements of our civilisation, also in areas such as sports science, medicine, nutrition, building, transport, and textiles (Maglischo, 2003; O’Connor and Vozenilek, 2011). A factor that has greatly contributed to new swimming records is the activity of the International Swimming Federation that in developing the competition rules remains open to new solutions concerning swimming techniques, starts, flip turns, swimmers’ gear and swimwear, swimming pool equipment, and makes sure that the final performance is determined by human skills and the quality of training rather than technology (FINA, 2009).

The freestyle swimming events that have been analysed to compare men’s and women’s performances have revealed their convergence. The table below shows that the longer the swimming distance, the smaller the difference between male and female results. According to Chatterjee and Laudato (1996), the explanation of why the difference between women and men decreases as the distance grows longer should be sought in physiological and morphological features.

According to Chatterjee and Laudato, the explanation of why the difference in swimming results between women and men decreases as the distance grows longer should be sought in physiological and morphological features. This phenomenon has been confirmed in track events where, the smallest margin of differences occurs between men and women in the marathon, while the greatest percentile differences occur in the long sprints, which depend on anaerobic glycolysis (Mageean et al., 2011; Fukuda et al., 2012). It thus seems that women have well developed aerobic metabolism, similar to men, while sprints both in track and fields and in swimming require the development of muscular strength and power which favours men because of the higher content of muscle tissue and better developed anaerobic metabolism, what has been confirmed by greater oxygen debt and higher lactate concentration following maximal efforts lasting from 30 to 60 seconds, what corresponds well in swimming to the 50 and 100m sprints (Mamer et al., 2011; Adamczyk, 2011). A higher fat content in female athletes may increase buoyancy, what can be considered a benefit in distance swimming. Owing to increased buoyancy and energy reserves from higher body fat content, women may soon approach the performance of men.

The comparison of men’s and women’s performances achieved at major swimming competitions additionally reveals more dynamic progression of results in the second group, thus confirming Chatterjee and Laudato’s findings (1996). While the competition of male athletes in elite sport has been observed for almost 100 years now, female athletes have been competing at the highest level for around 60 years. Their road to outstanding performances has been made much shorter by the lessons learnt from men’s sports and due to the growing interest of scientific community in female athletes.

A higher rate of improvement in women’s sport performances is probably the reason why many predictions of results for female athletes are so optimistic (Arellano et al. 1994, Busso and Thomas 2006, Silva et al. 2007). The predictions based on mathematical models (table 1) which show that women will outperform men in the future seem rather improbable. Following this line of reasoning and for a longer period of prediction one might expect that athletes’ times would equal zero at some point in the future, regardless of the length of the distance (Péronnet and Thibault, 1989).

Trying to predict swimmers’ performance, Heazlewood (2006) has analysed eleven regression models for each swimming event separately and then used the highest coefficient of determination to select the model with the best fit to the empirical data, but even then deviations from the actual results could not be avoided.

They were greater, the longer the swimming distance was. In attempting to prepare this type of a prediction the fact-based premises underlying athletic performance should be used more broadly, rather than trusting statistical computations. According to Pyne’a et al. (2004), the most reliable approach to predicting the winners of the Olympic Games is to monitor athletes’ times recorded in the year preceding the Games or during competitions organized by the national federation immediately before the Olympics.

The analysis of previous performance of freestyle swimmers and their prediction have revealed a number of interesting tendencies within separately examined results of men and women, as well as regarding the relations between them. Women’s results improve more dynamically compared with men’s. Moreover, the difference between women’s and men’s times is smaller, the longer the swimming distance. As both male and female athletes tend to compete more and more vigorously within their groups, the gap between the gold medallist and the last finisher in the final is constantly decreasing, what provides the best evidence that this sport discipline continues to develop.

## Figures and Tables

**Table 1:** The predicted performances of male and female freestyle swimmers for the 2012 Olympic Games in London based on a linear regression (LR), a non-linear regression (NR) and the time series data (TS). Developed by the authors

Event	Sex	Place	LR	R^2	NR	R^2	TS	φ^2^
50m	♀	1	23.85	0.880	23.86	0.880	23.84	0.190
		8	24.46	0.880	24.47	0.880	24.49	0.197
		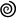 1÷8	24.18	0.876	24.19	0.977	24.15	0.110
	♂	1	21.47	0.850	21.47	0.850	21.49	0.310
		8	21.62	0.850	21.63	0.840	21.59	0.133
		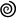 1÷8	21.47	0.971	21.47	0.971	21.46	0.138

100m	♀	1	50.87	0.866	51.22	0.872	50.91	0.159
		8	51.88	0.820	52.31	0.800	51.92	0.103
		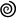 1÷8	48.64	0.882	48.92	0.872	51.46	0.174
	♂	1	45.78	0.910	46.03	0.910	45.83	0.100
		8	46.59	0.910	46.88	0.938	46.66	0.056
		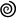 1÷8	46.25	0.938	46.51	0.938	46.19	0.060

200m	♀	1	1:53.87	0.786	1:54.05	0.797	1:57.92	0.350
		8	1:54.62	0.670	1:54.96	0.797	2:01.02	0.274
		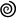 1÷8	1:54.25	0.796	1:54.49	0.797	1:59.47	0.380
	♂	1	1:42.01	0.878	1:42.16	0.878	1:45.35	0.225
		8	1:42.86	0.790	1:43.39	0.820	1:48.46	0.350
		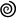 1÷8	1:43.24	0.878	1:43.47	0.878	1:47.08	0.290

400m	♀	1	3:47.15	0.789	3:50.12	0.833	3:45.92	0.327
		8	3:48.68	0.760	3:52.08	0.833	3:48.76	0.159
		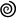 1÷8	3:47.15	0.821	3:50.12	0.833	3:47.19	0.177
	♂	1	3:30.17	0.897	3:31.72	0.897	3:33.75	0.115
		8	3:32.70	0.850	3:34.76	0.880	3:36.96	0.114
		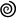 1÷8	3:31.78	0.897	3:33.57	0.897	3:35.62	0.110

800m	♀	1	8:04.42	0.791	8:05.45	0.697	8:04.38	0.070
		8	8:12.23	0.690	8:14.06	0.850	8:12.29	0.345
		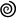 1÷8	8:08.15	0.794	8:09.61	0.697	8:08.10	0.370
1500m	♂	1	13:40.74	0.861	13:49.21	0.861	13:40.81	0.178
		8	13:54.53	0.800	14:06.30	0.820	13:54.45	0.162
		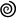 1÷8	13:49.59	0.861	13:59.03	0.861	13:49.56	0.156

R^2 – determination coefficient; ϕ – adjustment coefficient

**Table 2:** The performances of male and female freestyle swimmers – gold medallists at the Beijing Olympic Games in 2008

Event	Women	Men	Difference, s	Difference, %
50 m	00:24,06	00:21,30	00:02,76	11,47
100 m	00:53,12	00:47,21	00:05,91	11,13
200 m	01:54,82	01:42,96	00:11,86	10,33
400 m	04:03,22	03:41,86	00:21,36	8,78
800 m	08:24,54	07:47,11^*^	00:37,43	7,42

* best men’s 800m result in 2008 (http://swimnews.com)
